# From pulp to cementum: 3D visualization of soft and hard dental tissues using different ex vivo nano‐CT contrast‐enhancement techniques

**DOI:** 10.1111/iej.14260

**Published:** 2025-05-23

**Authors:** Torben Hildebrand, Håvard Jostein Haugen, Mario Romandini, Gianluca Plotino, Liebert Parreiras Nogueira

**Affiliations:** ^1^ Department of Biomaterials, Institute of Clinical Dentistry, Faculty of Dentistry University of Oslo Oslo Norway; ^2^ Perio‐Implant Innovation Center, Institute for Integrated Oral, Craniofacial and Sensory Research – National Clinical Research Center of Stomatology, Ninth People's Hospital Shanghai Jiao Tong University School of Medicine Shanghai China; ^3^ Private Practice Grande Plotino & Torsello Studio di Odontoiatria Rome Italy; ^4^ Oral Research Laboratory, Institute of Clinical Dentistry, Faculty of Dentistry University of Oslo Oslo Norway

**Keywords:** cementum, contrast‐enhanced nano‐CT, dental pulp, Lugol's iodine, PTA, soft tissue imaging

## Abstract

**Aim:**

To determine the effect of two contrast‐enhancement strategies in nano‐computed tomography (nano‐CT) imaging on the contrast‐to‐noise ratio (CNR) of various dental tissues, including pulp, dentine and cementum, with the goal of enhancing the visibility of dental soft tissues to a level not yet reported in laboratory nano‐CT imaging.

**Methodology:**

Ten sound human third molars underwent decalcification and subsequent treatment with Lugol's iodine (*n* = 5, Group 1) or phosphotungstic acid (PTA) treatment without prior decalcification (*n* = 5, Group 2) for contrast enhancement. Imaging was performed using the laboratory nano‐CT system Skyscan 2211 and the synchrotron radiation for medical physics (SYRMEP) beamline. CNRs were measured for pulpal tissue, dentine and cementum and nano‐CT images were compared with classical histology, scanning electron microscopy (SEM) and energy‐dispersive X‐ray spectroscopy (EDXS).

**Results:**

Group 1 significantly enhanced the contrast of pulp tissue, resulting in a 168.2% increase due to decalcification and an additional 148.7% increase after Lugol's iodine treatment. Dentine exhibited higher contrast in Group 2, whereas cementum showed similar contrast across both groups. Laboratory nano‐CT enabled the visualization of detailed soft tissue structures, including nerves, blood vessels and odontoblasts within the pulp, but cementocytes remained invisible.

**Conclusions:**

Decalcification followed by Lugol's iodine treatment was superior for enhancing soft tissue contrast, especially for pulp visualization. PTA without decalcification yielded better contrast for dentine and facilitated the visualization of attached soft tissues, such as periodontal ligament and predentine. These findings provide insights into selecting the most appropriate protocol to optimize nano‐CT imaging for specific dental tissue analyses, including the pulp.

## INTRODUCTION

In recent years, micro‐computed tomography (micro‐CT) has revolutionized endodontic research, complementing traditional histological analysis. The primary advantages of micro‐CT are its non‐destructive nature, eliminating the need for irreversible sample destruction and its ability to provide three‐dimensional visualization (Stauber & Müller, 2008). A limitation of micro‐CT has been its applicability, which was restricted to hard tissues, as soft tissues (e.g. dental pulp and periodontal ligament) lack sufficient X‐ray attenuation and appear ‘transparent’ (Ruegsegger et al., 1996). However, recent advancements have introduced methods enabling the visualization of soft tissues, with contrast‐enhanced micro‐CT (CE micro‐CT) (Dawood et al., 2021, 2022; Dawood & De Bakker, 2020; Descamps et al., 2014; Doost & Arnolda, 2021; Dunmore‐Buyze et al., 2014; Kampschulte et al., 2013; Lombardi et al., 2014; Metscher, 2009; Rivera‐Quiroz & Miller, 2022) and phase‐contrast imaging (Lee et al., 2024; Momose et al., 1996; Saccomano et al., 2024) emerging as two prominent techniques.

Contrast‐enhanced micro‐CT relies on chemically altering the x‐ray attenuation of a sample by incorporating contrast agents. These agents, enriched with high atomic number elements, enhance the contrast in absorption‐based x‐ray imaging. The phase‐contrast imaging technique (Mizutani & Suzuki, 2012), on the other hand, is based on the phase shift of x‐rays as they pass through a structure, offering a sensitivity that may be several orders of magnitude greater than absorption for light elements, such as hydrogen, carbon, nitrogen and oxygen. This property makes phase‐contrast imaging particularly effective for visualizing biological soft tissues (Momose et al., 1996). However, despite its advantages, phase‐contrast imaging requires synchrotron facilities, which limits accessibility (Davis & Elliott, 2003; Wu et al., 2017). Laboratory micro‐CT systems, on the other hand, are widely available and provide imaging capabilities comparable to synchrotron‐based systems in terms of submicrometre voxel resolutions (Attwood, 2006; Ritman, 2011).

A limited number of protocols have been proposed for CE micro‐CT applications in dental research so far. A recent study by De‐Deus et al. (De‐Deus et al., 2021) applied Lugol's iodine to enhance the visibility of dental pulp soft tissues. This approach achieved superior measurement accuracy compared to traditional histological techniques. However, the protocol did not allow for ultrastructural tissue evaluation. In a previous attempt, ultrastructural features of dental tissues were visualized using CE nano‐CT, which can be defined as a form of micro‐CT with voxel sizes below 1 μm, specifically 0.5 μm. This technique focused on small areas of the tooth and employed phosphotungstic acid (PTA) for contrast enhancement (Hildebrand et al., 2021). While this approach successfully highlighted soft tissue layers, it failed to show ultrastructure, individual cells and finer soft tissue details (Hildebrand et al., 2021).

Both strategies employed contrast enhancement of partially calcified tissues, necessitating strong soft tissue contrast to counterbalance the high radiopacity of dentine. An increased X‐ray tube power was required to ensure sufficient penetration, which enlarges the focal spot and degrades image resolution and detail for soft tissue structures (Rueckel et al., 2014). A promising modification of the protocol used by De‐Deus et al. (2021) involves complete tissue decalcification followed by Lugol's iodine treatment, thus decreasing overall attenuation and simultaneously increasing soft tissue contrast through iodine enrichment, as previously shown in bone (Hildebrand, Ma, Heyward, et al., 2024) and mouse mandibles (Hildebrand, Humphris et al., 2024).

The aim of the present study was to compare two strategies for enhancing contrast in dental tissues using high resolution nano‐CT: (1) complete decalcification of mineralised tissues followed by Lugol's iodine contrast enhancement and (2) direct soft tissue contrast enhancement using PTA without prior decalcification. Synchrotron radiation micro‐CT (SRμCT) was used as the reference standard for comparison. Additionally, classical histology, scanning electron microscopy (SEM) and energy‐dispersive X‐ray spectroscopy (EDXS) were employed to corroborate the findings obtained by laboratory nano‐CT and SRμCT.

## MATERIALS AND METHODS

The manuscript of this laboratory study has been written according to the Preferred Reporting Items for Laboratory Studies in Endodontology (PRILE) 2021 guidelines (Nagendrababu et al., 2021) and the flowchart is summarized in Figure S1.

### Tooth specimens

Ten sound human third molars were collected from healthy adult donors after obtaining written informed consent. The study was approved by the Regional Ethical Committee (REK 2024/737978). After extraction, the teeth were fixed in 4% buffered paraformaldehyde (PFA) in 50 mL centrifuge tubes at 7°C for 5 days and then stored in phosphate‐buffered saline (PBS) at 7°C until further processing.

The samples were divided into two groups:

- Group 1: Decalcification followed by Lugol's iodine contrast enhancement (*n* = 5).
- Group 2: PTA contrast enhancement (*n* = 5).

**FIGURE 1:**
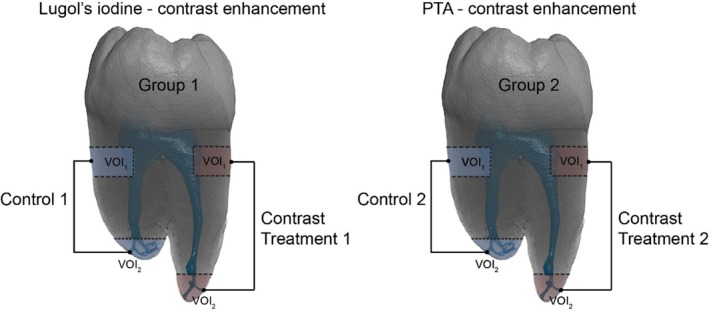
Sampling of molars for Lugol's iodine (Group 1) and PTA (Group 2) contrast enhancement. Samples were obtained from the coronal root third and the root apex. Controls were treated by the same protocol, excluding the exposure to the contrast agent.

Each tooth was sectioned using a water‐cooled micro bandsaw (MBS 240/E, PROXXON GmbH, Germany) to isolate two cross‐sections from the coronal third of the root (VOI_1_) and two from the apical third (VOI_2_) (Figure 1). In Group 1, tooth sectioning was performed after decalcification. Control specimens were additionally prepared without applying contrast agents for baseline comparison.

### Contrast‐enhancement protocols

**FIGURE 2:**
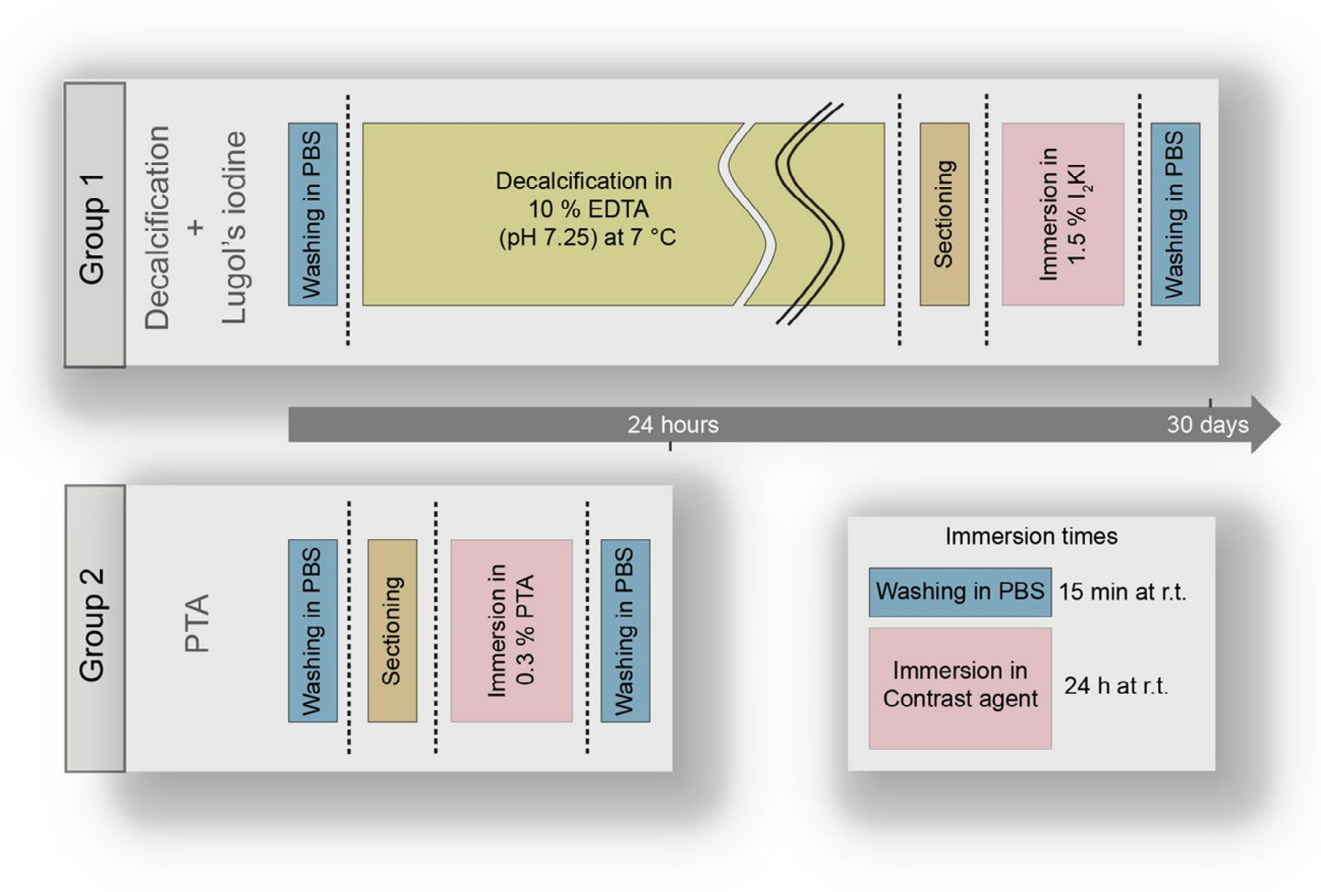
Preparation protocols for Lugol's iodine (Group 1) and PTA (Group 2) contrast enhancement of dental samples. All steps were performed on a rocking table at room temperature (r.t.), if not indicated differently.

Samples from Group 1 were first decalcified in 10% EDTA (pH 7.25) at 7°C. Then, they were immersed in 0.5% iodine (Sigma‐Aldrich, Saint Louis, MO 63103, USA) and 1% potassium iodide (Sigma‐Aldrich) in water. Samples from Group 2 were directly immersed in 0.3% PTA (Sigma‐Aldrich) in water. Both protocols are shown in Figure 2. Control samples followed identical preparation protocols, omitting specific staining steps (‘Immersion in 1.5% I_2_KI’ for Group 1, ‘Immersion in 0.3% PTA’ for Group 2).

All samples were then embedded in paraffin wax following a sequential preparation process:

1. Dehydration in graded ethanol solutions (30%–100%) in 10% increments, with each step lasting 15 min at room temperature.
2. Clearing with xylene three subsequent times, for 20, 20 and 45 min, respectively.
3. Wax Incubation in paraffin (Epredia, Kalamazoo, MI, USA) at 60°C, progressing through three types: Type 1 (30 min), Type 6 (30 min), Type 9 (30 min).

Following wax incubation, the samples were promptly removed and solidified for nano‐CT mounting.

### Synchrotron radiation micro‐CT (SRμCT)

Specimens from both groups, including controls from both VOIs, were scanned at the Synchrotron Radiation for Medical Physics (SYRMEP) beamline at the Elettra Synchrotron Laboratory (Trieste, Italy). The scanning parameters were optimized based on the contrast‐enhancement technique:

- Group 1 (Lugol's iodine, *n* = 5):
  ￮ Filter: 0.5 mm silicon.
  ￮ Energy: 16.7 keV.
  ￮ Exposure time: 50 ms.
  ￮ Projections: 1800 over 180°.
- Group 2 (PTA, *n* = 3):
  ￮ Filter: 1.0 mm aluminium and 1.5 mm silicon.
  ￮ Energy: 23.3 keV.
  ￮ Exposure time: 600 ms.
  ￮ Projections: 2400 over 180°.

All scans achieved a pixel size of 0.9 μm. A sample‐to‐detector distance of 100 mm was maintained for all specimens.

Reconstruction of the acquired projections was performed using the SYRMEP Tomo Project (STP) (Brun et al., 2017) (version 1.6.2) with a filtered back projection (FBP) algorithm incorporating a Shepp‐Logan filter in 32‐bit depth. Further visualization and analysis were performed using Dragonfly 3D World software (Comet Technologies Canada Inc., Montréal, Canada, version 2024.1, Build 1601).

### Contrast enhancement assessment

Scans obtained via SRμCT were analysed to evaluate the contrast‐to‐noise ratio (CNR) of key dental tissues: dental pulp and dentine in VOI_1_ and cellular cementum in VOI_2_. Measurements were performed both without (control) and with contrast enhancement.

For each scan, grey values of tissues of interest and background were measured in five spherical volumes of 100 μm in diameter. The CNR was calculated using Equation 1, incorporating separate noise contributions for the tissue of interest and the background as described in (Shahmoradi et al., 2016)

(1) $${\mathrm{CNR}}_{T}\;\left(1\right)=\frac{\left|{\bar{\mathrm{GV}}}_{T}-{\bar{\mathrm{GV}}}_{\mathrm{Bkg}}\right|}{\sqrt{{\mathrm{SD}}_{T}^{2}+{\mathrm{SD}}_{\mathrm{Bkg}}^{2}}},$$

where ${\mathrm{GV}}_{T}$ is the grey value of the tissue of interest and ${\mathrm{GV}}_{\mathrm{Bkg}}$ the grey value of the surrounding air. The divisor indicates the standard deviation (SD) of the grey values in the tissue and in the background.

The impact of contrast enhancement was evaluated by calculating the percentage increase in CNR for both Lugol's iodine (Group 1) and PTA (Group 2). For Group 1, the contrast enhancement was determined by comparing the CNR after decalcification and contrast enhancement (${\mathrm{CNR}}_{T}$) to the CNR before contrast enhancement (${\mathrm{CNR}}_{C}$). For Group 2, the contrast enhancement was calculated by comparing the CNR after contrast enhancement (${\mathrm{CNR}}_{T}$) to the CNR before contrast enhancement (${\mathrm{CNR}}_{C}$), without decalcification. The contrast enhancement was calculated using Equation 2:

(2) $$\text{Contrast enhancement}\;\left(\%\right)=\frac{{\mathrm{CNR}}_{T}-{\mathrm{CNR}}_{C}}{{\mathrm{CNR}}_{C}}∙100\%.$$

The same equation was also used to evaluate the change in contrast due to decalcification, using the controls from Group 1 (decalcified) and Group 2 (calcified) as reference CNR values.

### Nano‐computed tomography (nano‐CT)

Samples from each group were scanned (*n* = 1) using a nano‐CT device (Skyscan 2211 Multiscale X‐ray Nano‐CT System, Bruker Belgium SA, 2550 Kontich, Belgium). The system utilized a 20–190 kV tungsten X‐ray source and a dual detection set‐up comprising an 11‐megapixel cooled 4032 × 2670‐pixel CCD camera and a 3‐megapixel (1920 × 1536‐pixel) CMOS flat panel.

For specimens obtained from the tooth and prepared as detailed in Figure 2, scan parameters were optimized for each group, as summarized in Table 1.

**TABLE 1:** Scanning parameters for laboratory micro‐CT (Skyscan 2211).

Scanning parameter	Group 1: Lugol's iodine		Group 2: PTA	
	VOI_1_	VOI_2_	VOI_1_	VOI_2_
Filter	No filter	No Filter	0.125 mm Al	0.125 mm Al
Acceleration Voltage [kV]	40	50	50	60
Tube current [μA]	290	250	230	180
Exposure time [ms]	800	750	1300	1300
Scanning time	2 h 22 min	2 h 33 min	3 h 52 min	3 h 52 min

Scanning was performed utilizing the CCD camera with a rotation step of 0.17° over 360°, achieving a voxel size of 0.9 μm.

Nano‐CT projections were reconstructed using NRecon software (version 1.7.4.6) provided by the system, applying the fast hierarchical back‐projection (FHBP) algorithm, with a beam hardening correction of 30% and ring artefact correction of 12. The reconstructions were output in 16‐bit depth and subsequently visualized and analysed using Dragonfly software (Object Research Systems (ORS), Montréal, Canada, version 2024.1).

### Histology

Paraffin‐embedded samples from Group 1 were re‐embedded in base moulds using paraffin (Type 9; Epredia, Kalamazoo, MI 49008, USA) to ensure proper orientation. Sections of 7 μm thickness were obtained with a Leica RM2255 rotary microtome (Leica Biosystems Nussloch GmbH, 69 226 Nussloch, Germany) and mounted on Superfrost positively charged glass slides. The slides were baked, deparaffinized, rehydrated and stained with haematoxylin and eosin (H&E).

Samples from Group 2 were deparaffinized and subsequently embedded in Technovit® 7200 VLC resin (Kulzer GmbH, 63 450 Hanau, Germany). Resin blocks were glued onto slides and sectioned using a diamond band saw (310 CP; EXAKT Advanced Technologies GmbH, 22 851 Norderstedt, Germany). The sections were further ground and polished using a Micro Grinder 400 CS (EXAKT Advanced Technologies GmbH, 22 851 Norderstedt, Germany) to a thickness of approximately 30 μm. Final slides were stained with H&E.

### Scanning electron microscopy (SEM) and energy‐dispersive x‐ray spectroscopy (EDXS) analysis

For Group 1, additional unstained paraffin sections prepared for histology were mounted on aluminium stubs with double‐sided carbon tape. The samples were examined using a Hitachi Analytical TableTop Microscope/Benchtop SEM TM3030 paired with EDS equipment (Bruker Nano GmbH, 12 489 Berlin, Germany). Imaging was performed at an acceleration voltage of 15 kV, with a working distance of 6800 μm and magnifications of 1–2 k. Elemental mapping for the iodine within the sample was conducted using EDXS, with acquisition times totalling 3 min. The analysis targeted the dental pulp, the pulp‐dentine interface and cellular cementum.

For Group 2, samples were embedded in Technovit® resin and then trimmed, ground and polished following the same protocol as described for the histology. Sections (600 μm‐thick) were mounted onto an aluminium stub with double‐sided carbon tape. SEM and EDXS analysis were performed under the same conditions as Group 1, focusing on tungsten mapping.

### Data analysis

CNR values for dental pulp, dentine and cementum were analysed using two‐way anova to assess treatment effects (decalcification followed by Lugol's iodine contrast enhancement and PTA contrast enhancement). Data normality was tested using the Shapiro–Wilk test. Post‐hoc Tukey's test was applied to identify pairwise differences, with significance levels set at *p* > .05 (ns), *p* ≤ .05 (*), *p* ≤ .01 (**), *p* ≤ .001 (***) and *p* ≤ .0001 (****).

To highlight significant differences between groups, annotated boxplots showing the mean, minimum and maximum values were created. All statistical analyses were performed using GraphPad Prism (version 10.2.0).

## RESULTS

### Assessment of contrast enhancement

**FIGURE 3:**
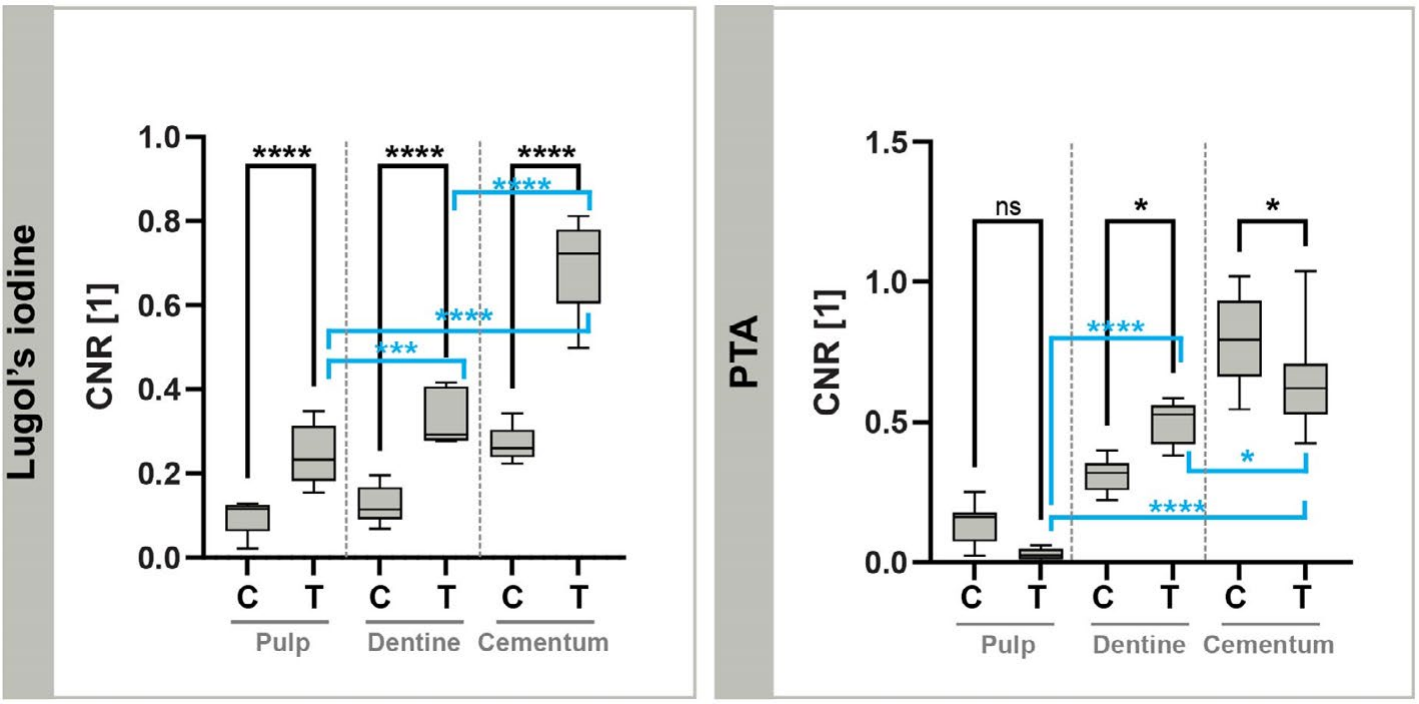
CNR values of contrast‐enhanced SRμCT with decalcification and Lugol's iodine (left) and PTA without decalcification (right) for dental pulp, dentine and cementum in the root apex. C = Control samples without contrast enhancement; T = treatment with contrast agent. Statistical analysis was done using the Tukey test and 2‐way anova, ‘ns’ *p* > .05, *p ⩽ .05, **p ⩽ .01, *** *p* ⩽ .001, ****p ⩽ .0001.

Contrast enhancement using Lugol's iodine after EDTA decalcification (Group 1) resulted in measurable increases in CNRs across the tissues examined, as reported in Table 2. Among the tissues, cellular cementum exhibited the highest contrast, followed by dentine, while pulp tissue displayed the lowest CNR values overall. The percentage increase in CNR from control (decalcified) samples to treated samples was 148.7% ± 79.8% for pulp tissue, 163.5% ± 68.6% for dentine and 159.7% ± 37.8% for cellular cementum. Decalcification alone altered contrast as follows: 168.2% for pulp, −63.4% for dentine and −66.0% for cellular cementum. The CNR values for control (C) and treated (T) samples across all measured VOIs are shown in Figure 3, which highlights significant contrast enhancements after Lugol's iodine treatment. Contrast differences among the treated tissue types also differed significantly.

**TABLE 2:** CNRs for pulp, dentine and cellular cementum after contrast enhancement with Lugol's iodine (Group 1) or PTA (Group 2) from SRμCT, according to Equation 1.

Contrast enhancement	Pulp	Dentine	Cementum
Lugol's iodine (Group 1)	0.24 ± 0.07	0.33 ± 0.07	0.70 ± 0.12
PTA (Group 2)	0.03 ± 0.02	0.50 ± 0.07	0.66 ± 0.19

PTA as a contrast agent without prior decalcification (Group 2) led to lower CNR in pulp tissue compared to Group 1. However, calcified dentine and cellular cementum in the apical region of the dental root showed higher CNR values (Table 2). Dentine treated with PTA showed higher CNRs than Lugol's iodine treatment, while cellular cementum showed slightly lower CNRs under the same conditions. The percentage change in contrast between untreated calcified tissues and calcified tissues treated with PTA was: −79.5% ± 67.5% for the pulp, 60.9% ± 14.4% for the dentine and −16.9% ± 5.2% for the cellular cementum. A decrease in CNR was observed in the pulp tissue, but it was not significant, while contrast changes in dentine and cementum were significant (*p* ⩽ .05).

### Contrast‐enhanced laboratory Nano‐CT of dental structures

#### Lugol's iodine (group 1)

**FIGURE 4:**
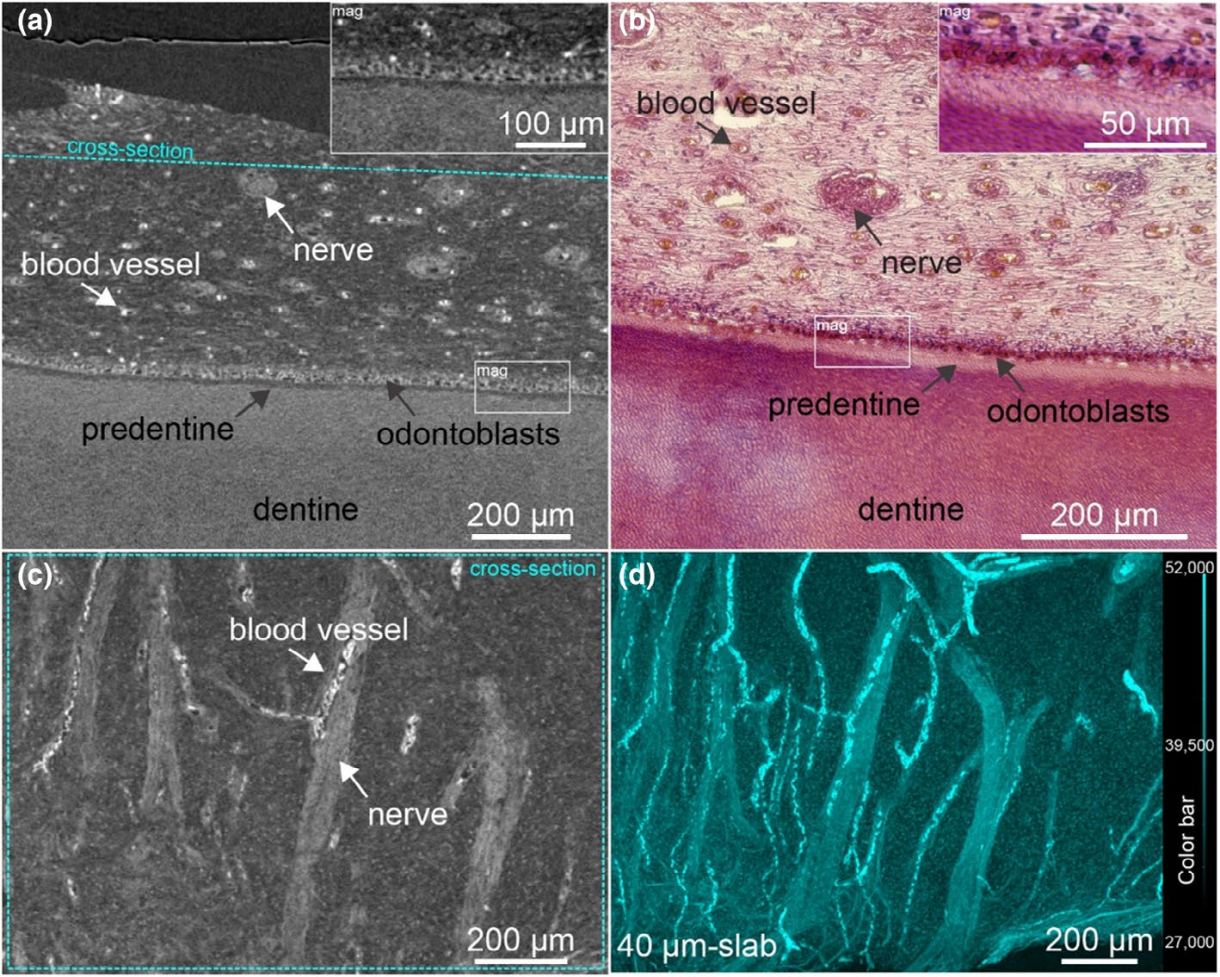
Dentine‐pulp interface imaged by nano‐CT using decalcification and contrast enhancement by Lugol's iodine and comparison with classical histology. (a) Nano‐CT cross‐section and (b) light microscopic section (H&E) from the coronal portion of the root of the same sample. (c) Nano‐CT cross‐section positioned parallel to the dentine showing blood vessels and nerves. (d) A slab of 40 μm thickness of the same section demonstrates the arrangement of blood vessels and nerves in accordance with depth.

Decalcification of dental tissues and contrast enhancement with Lugol's iodine provided a detailed visualization of the dental ultrastructure. In the coronal cross‐section of the root (VOI_1_), the network of blood vessels and capillaries became strongly enhanced, with the odontoblasts layer clearly visible along the dentine surface (Figure 4a). A lower radio‐translucent gap corresponding to predentine was observed between the odontoblasts and dentine. At higher magnification, individual odontoblasts were rudimentally distinguishable (Figure 4a, mag). Classical histology using H&E staining from the same sample confirmed these observations (Figure 4b). Erythrocytes exhibited higher contrast compared to the vascular walls. In comparison, nerves within the dental pulp displayed lower contrast than erythrocytes, making it possible to distinguish them from blood vessels (Figure 4a–c). Cross‐sections parallel to the dentine revealed the spatial relationship between blood vessels and nerves, with blood vessels encircling and penetrating the nerves (cyan structures in Figure 4d). Surrounding fibroblasts were visible as indistinct cell clusters, and the extracellular matrix showed no significant contrast enhancement.

**FIGURE 5:**
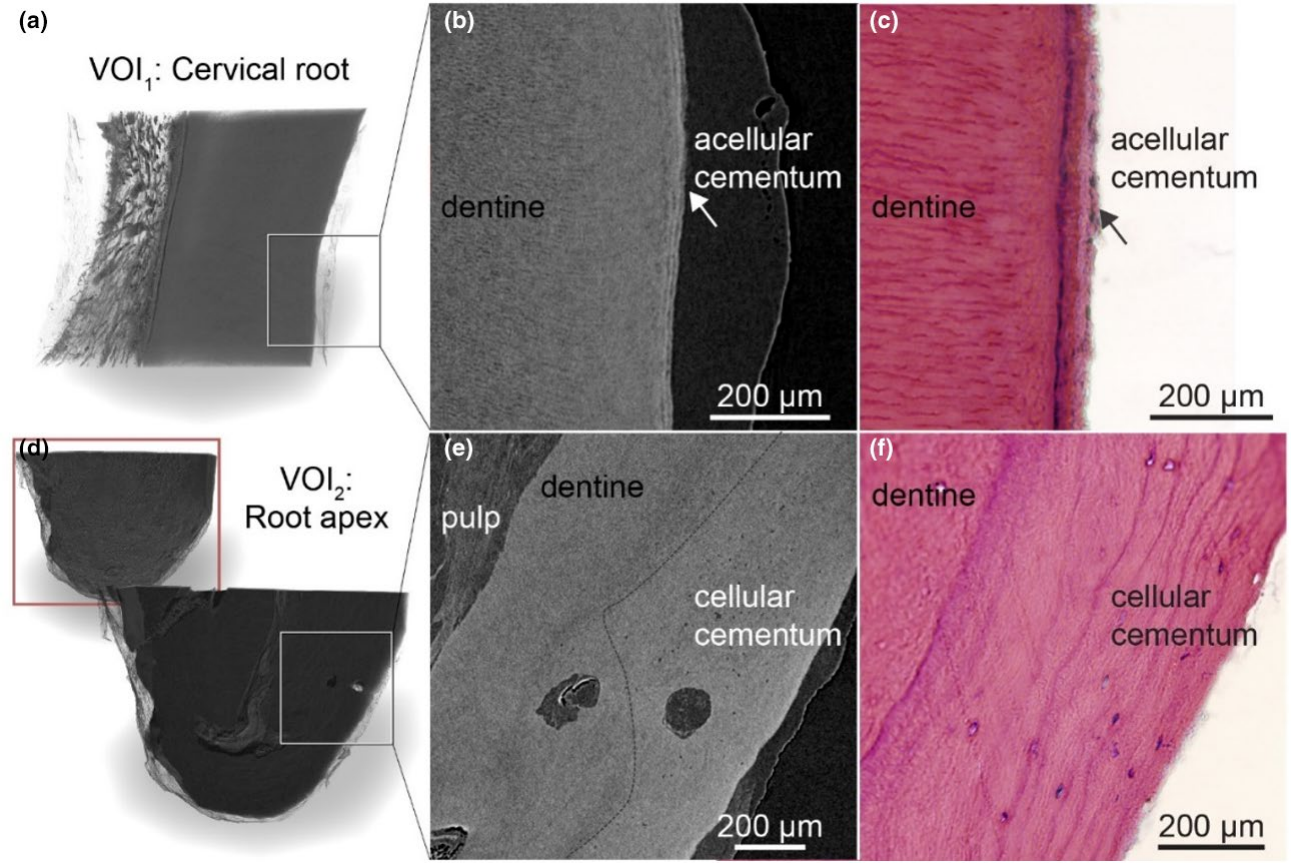
Acellular and cellular cementum were visualized by CE nano‐CT and H&E‐stained sections from the same sample. (a) Acellular cementum imaged by nano‐CT and preparation protocol using Lugol's iodine as a contrast agent in 3D and (b) in a representative cross‐section. (c) Corresponding acellular cementum by classical histology (H&E staining). (d) Cellular cementum by nano‐CT imaging in 3D, (e) in a representative cross‐section and (f) by classical histologic sectioning and H&E staining.

The acellular cementum was distinctly visible in the nano‐CT images in the apical region, with well‐delineated resting lines (Figure 5a). However, periodontal ligament (PDL) and cementoblasts were absent (Figure 5b). In the most apical portion, the cellular cementum and dentine exhibited minor differences in grey values, confirming the contrasts observed in SRμCT (Figure 5e). A dashed line demarcates the boundary between cementum and dentine. While cementocytes were identifiable in the corresponding H&E section (Figure 5f), they were not discernible in nano‐CT images.

#### PTA (group 2)

**FIGURE 6:**
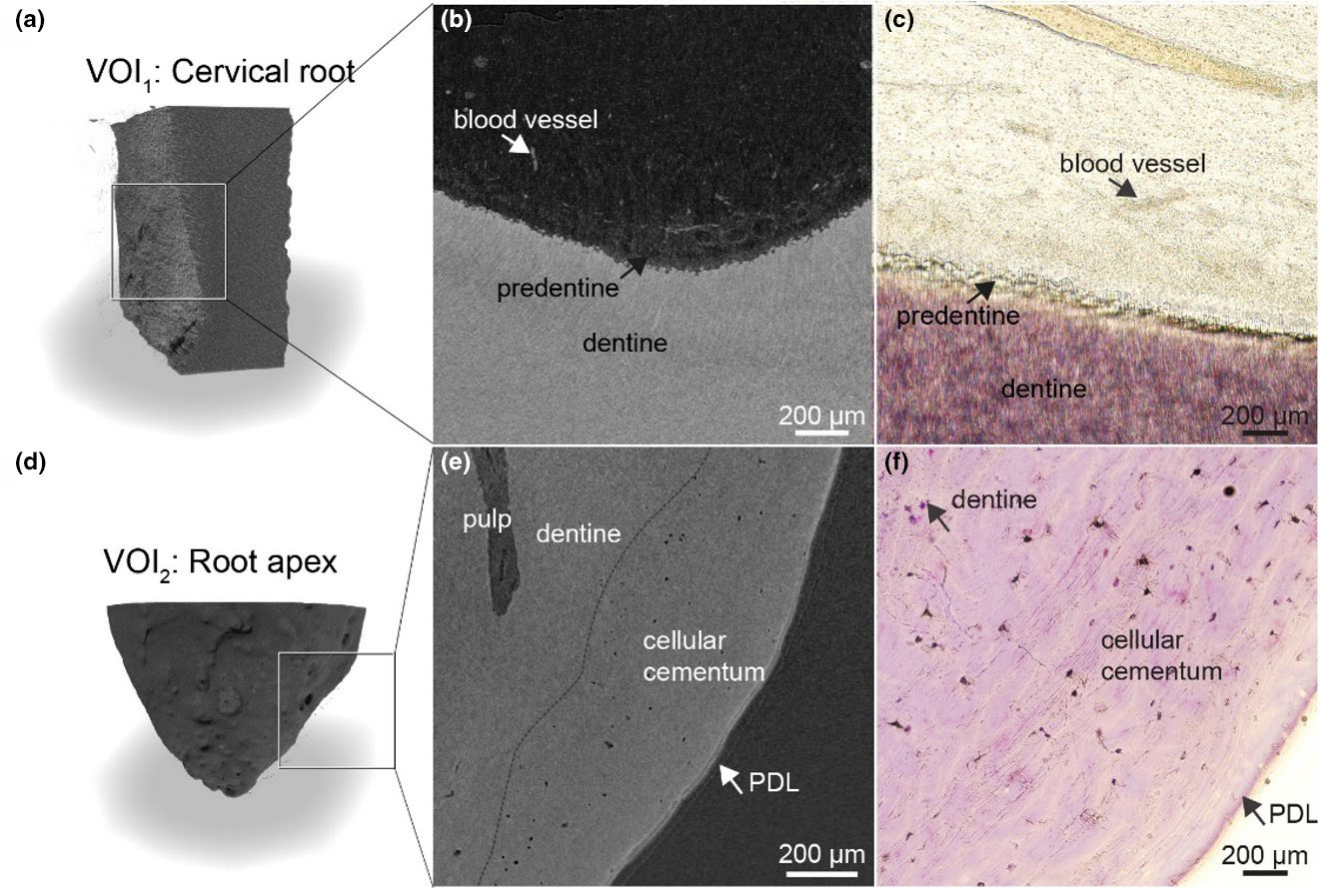
Dentine‐pulp interface and dental apex imaged by nano‐CT imaging using contrast enhancement by PTA and comparison with classical histology (H&E staining). (a) Pulp‐dentine interface imaged by nano‐CT and preparation protocol using PTA as a contrast agent in 3D and (b) in a representative cross‐section. (c) Corresponding tissue by classical histology (H&E staining). PTA enhanced the network of blood vessels and capillaries in the pulp. (d) Cellular cementum is obtained through nano‐CT imaging in 3D, (e) in a representative cross‐section and (f) in a classical histologic sectioning and H&E staining. The grey values of dentine and cellular cementum are similar and PDL is strongly enhanced. Cellular elements are not visualized in detail in nano‐CT images. H&E sections from the same sample after nano‐CT imaging revealed the presence of the cells in the analysed tissues.

The native mineralised state of dental tissues combined with PTA contrast enhancement provided limited ultrastructural detail. Blood vessels were distinctly identified in the cervical portion of the dental root (VOI_1_) and the predentine layer was clearly visible (Figure 6). The dentine‐pulp interface displayed the characteristic contours of mineralised dentine. In the apical region (VOI_2_), dentine and cellular cementum exhibited similar grey values, rendering them indistinguishable by contrast (Figure 6e). However, the PDL attached to the cementum showed higher contrast, enhancing its visibility and differentiation from adjacent tissues.

### Contrast‐enhanced SRμCT of the dental pulp

**FIGURE 7:**
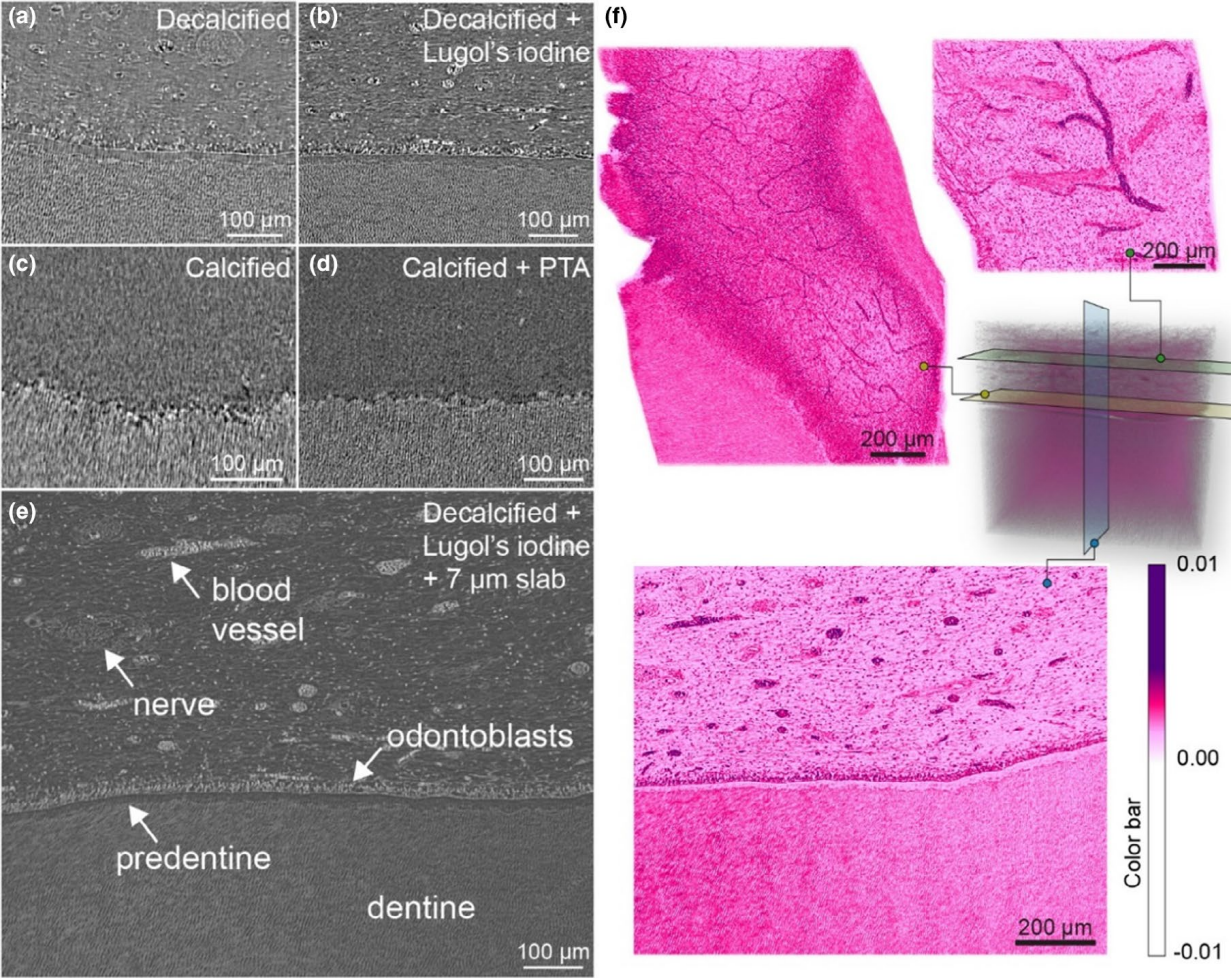
Different stages in contrast‐enhanced SRμCT of the dentine‐pulp interface. (a) Non‐treated and decalcified (control) and (b) decalcified and contrast‐enhanced (Lugol's iodine) cross‐section. (c) Calcified and (d) calcified and contrast‐enhanced cross‐section (PTA). (e) A 7‐μm slab of (b), further enhancing histological information. (f) Virtual histology of the decalcified and Lugol's iodine contrast‐enhanced sample. Artificial colouring, mimicking H and E‐staining, is applied. The network of blood vessels and soft tissue features can be examined in any orientation.

Cross‐sections obtained from SRμCT exhibited superior resolution compared to laboratory nano‐CT images. Applying Lugol's iodine in SRμCT significantly enhanced cell contrast within the dental pulp (Figure 7a,b). Slabs of 7 μm thickness, mimicking the histological section, further facilitated the identification of individual cells (Figure 7e). Artificial colouring and cross‐sections taken parallel to the pulp‐dentine interface revealed variations in the thickness and density of the capillary network (Figure 7f). Video S1 presents continuous cross‐sections through the pulp and the pulp‐dentine interface, highlighting the neural and capillary networks at varying distances from the dentine.

In PTA‐treated specimens, blood vessels within the pulp area became discernible compared to control samples (Figure 7c,d). However, other features, including fibroblasts, odontoblasts and nerves remained invisible alongside the calcified dentine.

### SEM and EDXS analysis

**FIGURE 8:**
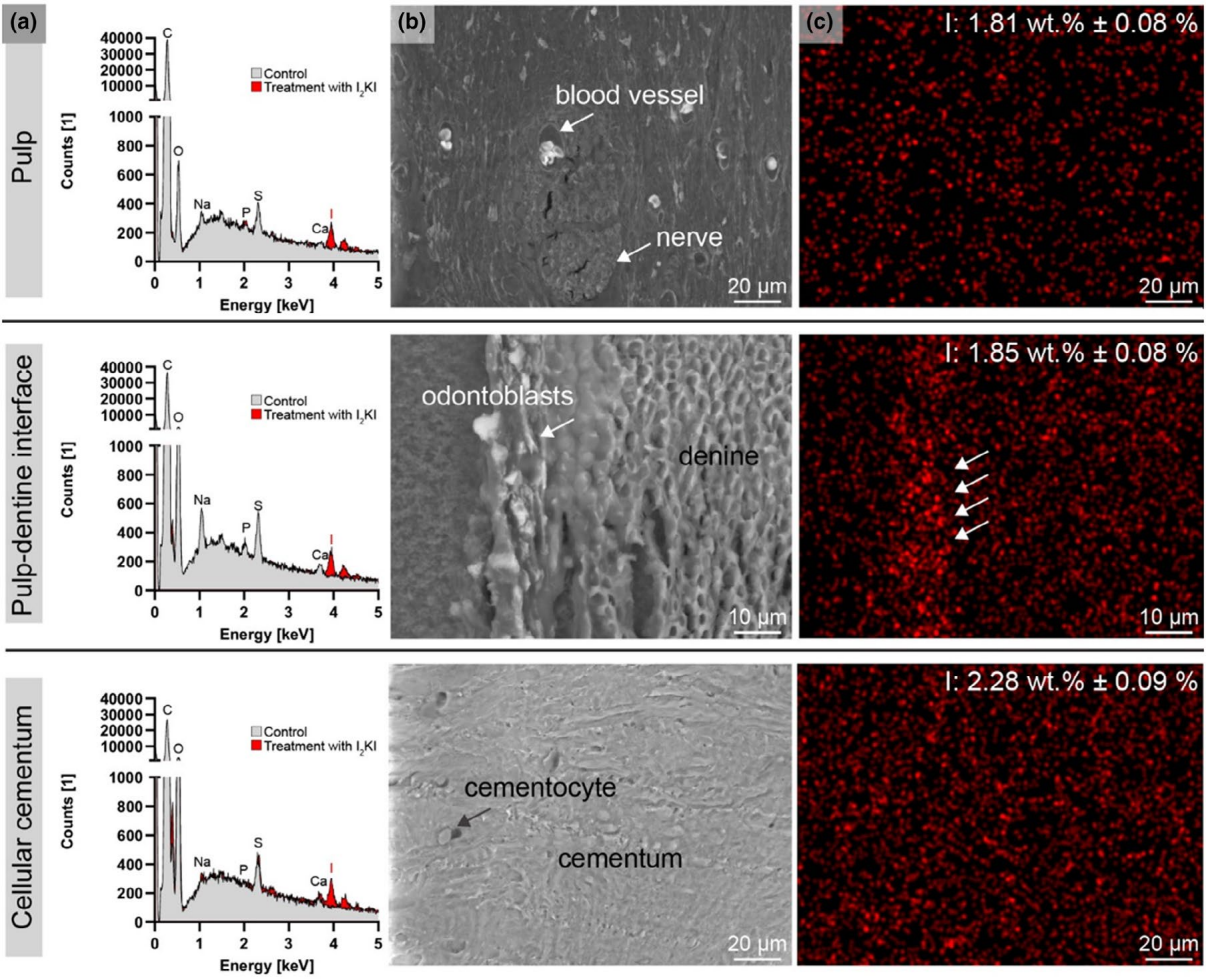
SEM‐EDXS of 7 μm‐thick sections from paraffin‐embedded dental samples, previously contrast‐enhanced with Lugol's iodine. (a) EDXS spectrum of pulp, pulp‐dentine interface and cellular cementum from untreated (control) and contrast‐enhanced samples. (b) SEM images from the selected dental regions and (c) iodine (I) map for images shown in (b).

**FIGURE 9:**
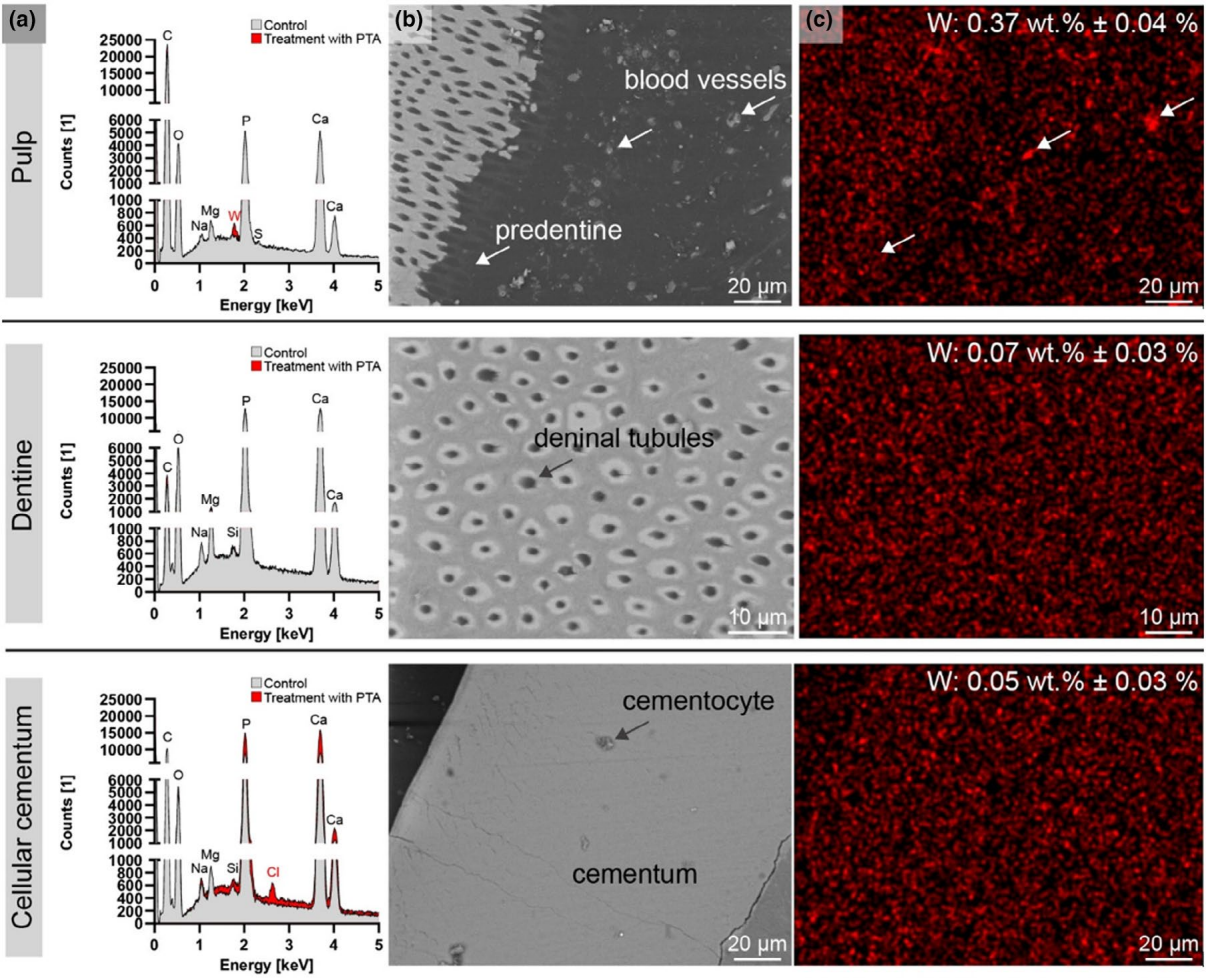
SEM‐EDXS of approximately 600 μm‐thick sections from Technovit®‐embedded dental samples, previously contrast‐enhanced with PTA. (a) EDX spectrum of pulp‐dentine interface, dentine and cellular cementum from untreated (control) and contrast‐enhanced samples. (b) SEM images from the selected dental regions and (c) tungsten (W) map for images shown in (b).

SEM/EDXS analysis of the nano‐CT scanned samples revealed the presence of the heavy metal iodine in Group 1 (Figure 8) and tungsten in Group 2 (Figure 9).

In Group 1, iodine concentrations were measurable, with clear peaks corresponding to iodine in the EDX spectrum, distinguishing the contrast‐enhanced samples from the non‐treated controls. SEM images of pulp tissue in iodine‐treated samples showed erythrocytes, nerves and surrounding cells, all contrast‐enhanced by iodine. Odontoblasts exhibited high conductivity in SEM images, and iodine appeared as agglomerates in the EDXS mapping.

Group 2 (PTA‐treated samples) demonstrated higher SEM image quality, attributed to their embedding, trimming and polishing in Technovit® resin, which exposed the dentinal tubules. In contrast to iodine‐enhanced samples, the pulp tissue in PTA‐treated samples was not generally enhanced for SEM imaging. However, tungsten was detectable in the blood vessels of the pulp, with slight agglomeration seen in the predentine (Figure 9c, arrows).

## DISCUSSION

To address the challenge of limited visibility of the soft tissue ultrastructure in teeth using micro‐ and nano‐CT, as well as the current contrast enhancement strategies, we present a comparison of two approaches aimed at improving ultrastructural tissue evaluation. These approaches build on previous attempts utilizing contrast‐enhanced CT (De‐Deus et al., 2021; Hildebrand et al., 2021) and include decalcification followed by treatment with Lugol's iodine as a novel strategy, as well as soft tissue contrast enhancement with PTA, without decalcification.

Contrast‐to‐noise ratio measurements indicated that decalcification before Lugol's iodine treatment is beneficial for pulpal tissues, dentine exhibited better contrast enhancement with PTA without decalcification, while cementum showed similar results for both protocols. Furthermore, both protocols resulted in increased tissue contrast compared to control samples, except for cellular cementum in the PTA protocol.

Phosphotungstic acid is known to function as both a decalcifying and contrast‐enhancing agent (Bonucci, 1967). However, dehydration steps during ethanol processing and embedding in paraffin may have led to a reduction in PTA concentrations, potentially lowering the contrast in nano‐CT images. Additional deparaffinising and embedding in Technovit® resin could have further decreased the concentration of the agent before EDX measurement, which showed low tungsten signals. Blood vessels, which were enhanced in nano‐CT images, also indicated tungsten in EDXS. However, the characteristic shape of the calcified dentine–predentine interface was highlighted to a greater extent compared with Group 1 or no contrast enhancement. Compared to a previous study utilizing PTA as a contrast agent (Hildebrand et al., 2021), the predentine was enhanced to a lesser extent. This could be due to several reasons: higher concentrations may have increased the binding of the contrast agent to the tissue; samples were contrast‐enhanced in PTA in ethanol, which could have affected the uptake of the contrast agent; samples were directly scanned after contrast enhancement and were not exposed to processing and embedding steps for paraffin mounting, which could have caused leakage of the contrast agent. Conversely, PTA was more robust against diffusion than iodine‐based contrast enhancement (Hildebrand et al., 2023), likely due to its higher molecular weight and hydrodynamic radius compared to Lugol's iodine (Schoborg et al., 2019). However, the low signal for tungsten in the tissues and the low contrast in nano‐CT support the assumption that the contrast enhancement with PTA in calcified tissue was compromised by subsequent processing, potentially underestimating the effectiveness of PTA contrast enhancement in this study. The presence of the pulp tissue in the samples was confirmed with H&E‐stained cross‐sections in light microscopy from the same samples after the scan, ruling out the absence of pulp. The selection of H&E staining for the samples is explained by its superior ability to present dental microanatomy and its good reproducibility (Widbiller et al., 2021).

For samples treated with Lugol's iodine, the decalcification process alone caused a CNR increase in pulpal tissue by 168.2%, while the contrast of dentine and apical cementum decreased. This can be explained by the decreased attenuation of the calcified tissues and the adjustment in the nano‐CT setup to a lower energy level for decalcified samples. However, contrast enhancement with Lugol's iodine additionally increased the contrast of the pulp by 148.7%, making this protocol an efficient strategy for enhancing soft tissue contrast along dentine.

Laboratory nano‐CT of the same samples supported the observations from SRμCT, allowing visualization of the dental soft tissues along dentine and cementum. In particular, the pulpal tissue showed nerves, blood vessels, individual cells in the pulp and odontoblasts aligned at the predentine. Different grey values allowed for the differentiation of erythrocytes and nerves in the dental pulp. Individual cells can be observed in the dental pulp; however, the clustered odontoblasts are optically difficult to separate. In SRμCT, the optical separation of the cells was improved, which can be explained by the unique beam qualities at synchrotron facilities enabling higher resolutions at the same voxel size. Acellular and cellular cementum appeared to have slightly higher contrast than dentine and could therefore be observed as lighter. However, individual cementocytes could not be observed. This observation is consistent with previous results of the same contrast enhancement protocol in bone, where osteocytes—analogous to cementocytes in their embedding in a collagenous and calcified matrix—were also not visible (Hildebrand, Ma, Heyward, et al., 2024).

Energy‐dispersive x‐ray spectroscopy measurements supported observations from nano‐CT imaging, confirming iodine in group 1, as the determining factor for contrast increase in nano‐CT images. Mapping the iodine in the dental ultrastructure was only possible to a limited extent. A stronger signal for iodine was noticeable in the region of the multilayer of odontoblasts, which corresponds with the enhanced contrasts in nano‐CT imaging for this structure, as illustrated in Figure 4. A general contrast enhancement with Lugol's iodine in tissues and redistribution in collagenous regions of the decalcified portions of the sample due to dehydration in ethanol (Hildebrand, Humphris et al., 2024) and paraffin embedding for mounting can explain the diffuse iodine signal in mapping.

The advantages and disadvantages of the utilized preparation methods can be summarized as follows: protocol 1 (Decalcification & Lugol's iodine Contrast enhancement) provided excellent visualization of cellular‐level soft tissue detail, easy manipulation of the sample, and a fast integration into a paraffin‐based sectioning routine for classical histology, while requiring additional preparation time due to decalcification and losing information about initial mineralisation; protocol 2 (PTA Contrast enhancement without decalcification) showed native degree of mineralisation and fast sample preparation, despite longer acquisition times, focal spot enlargement and difficult sample manipulation.

Limitations of the study lie in the voxel size of 0.9 μm, which was able to reveal cellular components and cells, but not to clearly separate individual cells in higher cell densities. However, the detail is superior to whole tooth scans or previously conducted scans at smaller voxel sizes but with higher tube powers. Another limitation remains in the unresolved binding properties of the contrast agents. PTA has been shown to enhance contrast in cells, including their nucleus and cell body in cell cultures (Hildebrand, Ma, Loca, et al., 2024); thus odontoblast processes could potentially be visualized. However, the affinity for collagen and dentine decreases the visibility of the entire cell. After dehydration in ethanol with Lugol's iodine, similar effects were observed (Hildebrand, Humphris et al., 2024) – redistribution of the contrast agent to collagenous volumes—impeding the unrestricted enhancement of the cellular portion in the decalcified dentine matrix. Addressing this knowledge gap is essential before drawing conclusions about tissue composition in relation to artificial colouring in virtual histology, with secondary modalities required for verification. Furthermore, it may be controversial that decalcification of the sample and contamination with heavy metals may qualify the contrast‐enhanced nano‐CT and proposed protocol as non‐invasive. Sample preparation must be adjusted considering subsequent analysis and contrast agent removal with sodium thiosulfate for iodine (Hopkins et al., 2015) or EDTA for PTA, which acts as a chelating agent (Nkuna et al., 2022; Sun et al., 2001; Zhang et al., 2021), should be considered.

Although micro‐CT has revolutionized endodontic research, it has significant limitations in visualizing soft tissues and microbial biofilms that may remain attached to root canal walls, particularly in complex anatomical regions, such as isthmuses, fins, anastomoses and accessory canals (Versiani et al., 2013). The present study emphasizes the significant relevance of CE nano‐CT in advancing endodontic research and, indirectly, clinical practice. Based on the current findings, researchers must select the most appropriate protocol to optimize nano‐CT visualization for specific dental tissue analyses, including the pulp. This capability has the potential to provide new insights into the pathogenetic processes of endodontic diseases, allowing for more precise diagnosis and treatment planning (Bhat et al., 2024; Donnermeyer et al., 2023; Rotstein & Simon, 2004). Contrast‐enhanced nano‐CT can be particularly valuable for exploring regenerative endodontic procedures, such as pulp revascularisation and stem‐cell‐based therapies, by assessing the quality of tissue regeneration, including vascularisation, cellular composition and integration with native structures. The results of the present study highlight the potential of combining decalcification with Lugol's iodine treatment as an innovative and promising approach in these applications. Furthermore, it offers the potential for quantitative analysis of tissue changes and pathologies, such as calcifications or biofilm progression. It provides a powerful platform for assessing the performance and interaction of endodontic materials, including root canal sealers and specifically bioactive cements by enabling the three‐dimensional visualization of their spatial distribution, interface with dentinal walls, and potential leakage pathways, the combined use of decalcification and Lugol's iodine treatment enhances contrast for soft tissues, allowing for the evaluation of remaining pulp tissue, periodontal structures, and their relationship to the applied materials. When combined with biomaterials, contrast‐enhanced nano‐CT without decalcification, but with stronger contrast enhancement, can significantly improve visualization, albeit at the cost of resolution and increased scan time. These attributes not only enhance the understanding of disease mechanisms and treatment efficacy but also hold promise for developing non‐invasive diagnostic approaches, potentially improving the management of complex endodontic cases and advancing regenerative and restorative therapies in clinical practice.

## CONCLUSION

Contrary to previous approaches in contrast‐enhanced micro‐CT of human dental tissues (De‐Deus et al., 2021; Hildebrand et al., 2021), the present study focused on an additional strategy to improve contrast in soft tissue imaging: decalcification followed by contrast enhancement with Lugol's iodine. At high resolution, this treatment effectively enhanced soft tissue contrast, especially for pulp visualization: odontoblasts, nerves, capillaries and periodontal ligament became visible at the ultrastructural level in laboratory absorption nano‐CT. Contrast enhancement with PTA without decalcification also enhanced soft tissue structures, but to a lower extent, while yielded better contrast for dentine and facilitated the visualization of attached soft tissues, such as periodontal ligament and predentine. While preparation in the calcified state is faster and less invasive, decalcification followed by contrast enhancement demonstrated superior soft tissue visualization and seamless integration into a classical histology routine, including paraffin sectioning. These findings provide insights into selecting the most appropriate protocol to optimize nano‐CT imaging for specific dental tissue analyses. The choice of optimal sample preparation for contrast‐enhanced nano‐CT needs to be finely adjusted according to the study's aim and subsequent analysis methods. However, this study demonstrated a superior and optimized strategy for visualizing soft dental tissues, including pulp, which is highly comparable to classical histological approaches.

## AUTHOR CONTRIBUTIONS

**Torben Hildebrand**: Writing—original draft, visualization, investigation, formal analysis. **Håvard Jostein Haugen**: Writing—Review and editing, conceptualisation, resources. **Mario Romandini:** Writing—review and editing. **Gianluca Plotino**: Writing–review and editing. **Liebert Parreiras Nogueira**: Writing—review and editing, supervision.

## FUNDING INFORMATION

We acknowledge Elettra Sincrotrone Trieste for providing access to its synchrotron radiation facilities (Project No. 20240522) and the financing by the EU Interreg Öresund‐Kattegat‐Skagerrak project ‘Hanseatic Life Science Research Infrastructure Consortium’ (HALRIC).

## CONFLICT OF INTEREST STATEMENT

The authors declare that they have no known competing financial interests or personal relationships that could have appeared to influence the work reported in this paper.

## ETHICS STATEMENT

Sound human third molars were collected from healthy adults as part of standard clinical procedures where extraction was necessary. Written consent was obtained from all patients, and the collection was approved by the Regional Ethical Committee (REK 2024/737978, Southeast Region, Norway).

## Supporting information


**Figure S1.** PRILE 2021 flowchart for the present study.

**Video S1.** Animated cross‐sections of the pulp and pulp–dentine interface, highlighting neural and capillary networks at varying distances from the dentine.

## Data Availability

The data that support the findings of this study are available from the corresponding author upon reasonable request.
