# Supplementary figures and images for: From pulp to cementum: 3D visualization of soft and hard dental tissues using different ex vivo nano‐CT contrast‐enhancement techniques
Source: Int Endod J. 2025 May 23;58(8):1228–42. doi: 10.1111/iej.14260 (PMC12254532; doi:10.1111/iej.14260)

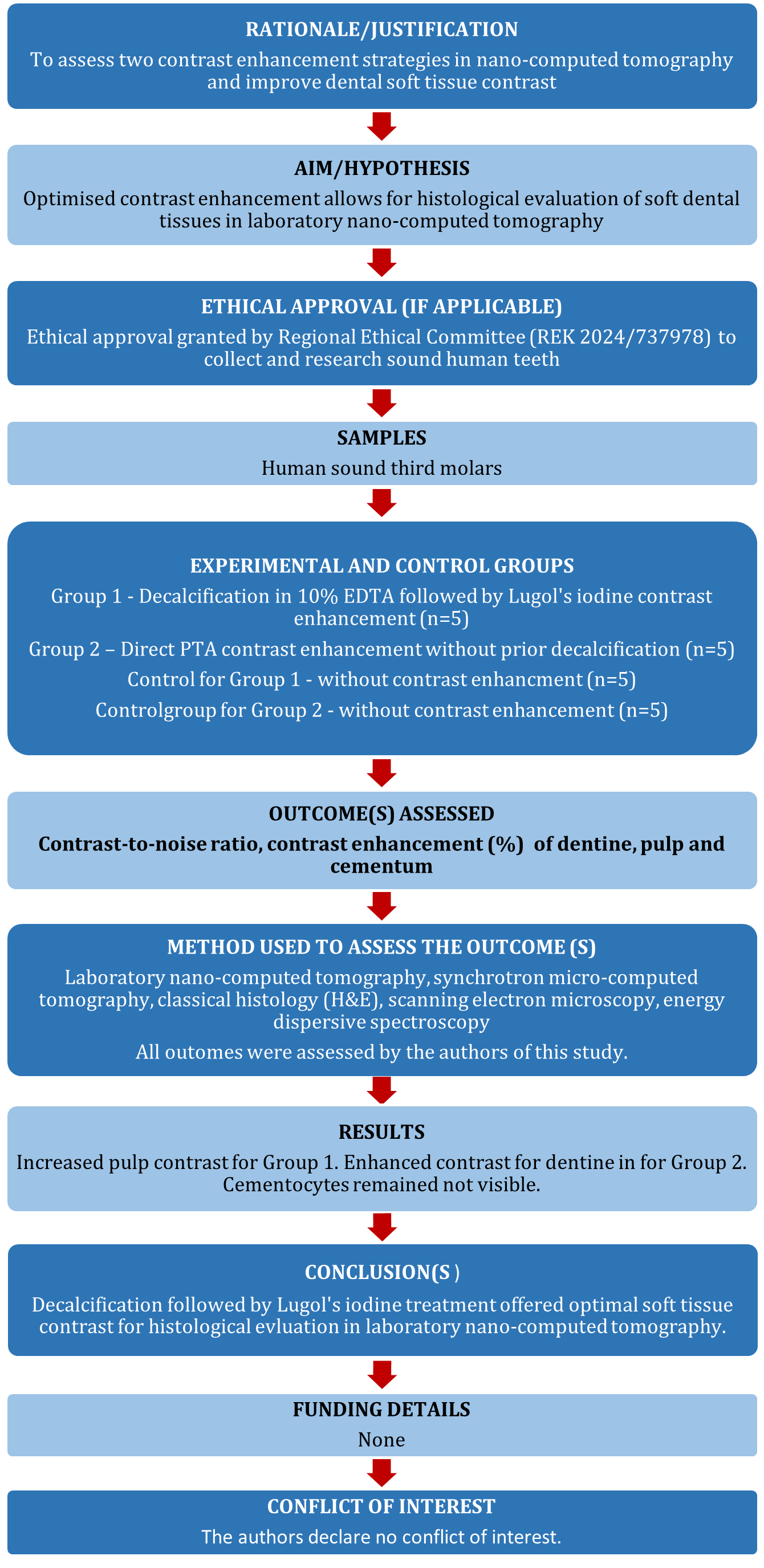

Supplement: Supplementary file 1 — Figure S1. PRILE 2021 flowchart for the present study. [file IEJ-58-1228-s002.png]
